# Parenting Practices and Psychosomatic Complaints Among Swedish Adolescents

**DOI:** 10.3389/ijph.2023.1606580

**Published:** 2023-12-21

**Authors:** Karina Grigorian, Viveca Östberg, Jonas Raninen, Sara Brolin Låftman

**Affiliations:** ^1^ Department of Public Health Sciences, Stockholm University, Stockholm, Sweden; ^2^ Department of Clinical Neuroscience, Karolinska Institutet (KI), Stockholm, Sweden; ^3^ Centre for Alcohol Policy Research, La Trobe University, Melbourne, VIC, Australia

**Keywords:** parenting practices, parental support, adolescence, psychosomatic complaints, two-wave data

## Abstract

**Objectives:** Parent-adolescent relationships play a crucial role in youth development. This study examines the associations between parenting practices (parental support, knowledge, and rule-setting) and psychosomatic complaints across middle and late adolescence.

**Methods:** The study utilised data from a Swedish national cohort (*n* = 3,678). Participants completed self-report questionnaires in 2017 (∼15–16 years) and again in 2019 (∼17–18 years).

**Results:** Parental support exhibited the strongest and most consistent inverse cross-sectional associations with psychosomatic complaints during both middle and late adolescence. Furthermore, increases in parental support and parental knowledge were associated with decreases in adolescent psychosomatic complaints. However, parental support and knowledge at age 15–16 were not prospectively associated with psychosomatic complaints at age 17–18.

**Conclusion:** These findings underscore the importance of ongoing parental engagement, particularly in terms of providing constant support, throughout middle and late adolescence.

## Introduction

Psychosomatic complaints are common among adolescents, particularly among girls and in older age groups [1–3]. They typically encompass a range of subjective psychological and physical health complaints, such as headache, stomach ache, and difficulties to fall asleep. This type of complaints has been shown to be indicative of stress [4] and is generally regarded as an indicator of the broader concept of “mental health problems” [5, 6]. Psychosomatic complaints are recognised as a public health concern in many countries [7], including Sweden [6].

Parent-adolescent relationships play a crucial role in adolescent development and are associated with multiple physical and mental health outcomes in both the short and long term [8, 9]. Previous research has also shown that adolescents with low-quality parent-adolescent relationships tend to experience more psychosomatic complaints [10, 11]. Nonetheless, it has been emphasised by a Lancet commission on adolescent health and wellbeing that despite the pivotal role of families and parents in the lives of most adolescents, “the paucity of rigorous research into family influences on adolescent health and wellbeing is a striking knowledge gap” [12] (p. 15). Furthermore, Morris et al. underscored in their paper that effective prevention and intervention strategies for addressing mental health challenges among adolescents are scarce, and given the potential utility of understanding the relationships between parenting and adolescent mental health for developing such strategies [13], there is a critical need for further knowledge in this area. Particularly, in the light of recent research indicating the enduring significance of parent-adolescent relationships in older age groups [13], there is a strong relevance in studying parenting factors related to mental health in older adolescents.

### Parenting Practices and Adolescent Health

The parent-adolescent relationship is closely intertwined with parenting practices [14]. Parenting practices are vital components of parenting and encompass specific behaviours through which parents perform their parental responsibilities, aimed at the child’s development [15]. Different parenting practices typically represent interconnected yet distinct facets of parental involvement [16, 17]. Numerous studies have highlighted the significant role of parenting practices in youth health [18, 19]. Moreover, parenting practices are considered dynamic processes, indicating that parents may vary in these practices over time and in different situations. This study focuses on three central parenting practices, namely parental support, knowledge and rule-setting, which embody intertwined yet unique dimensions of parental participation that may be associated with adolescents’ psychosomatic complaints.

Parental support serves as one of the most crucial parenting practices and an independently important source of social support throughout adolescence [20]. Parental support has consistently been associated with better adolescent adjustment and fewer mental health problems, including psychosomatic complaints [20–22]. Conversely, lower levels of perceived parental support and a lack of parental warmth have been linked to higher levels of mental health problems [23]. Furthermore, despite the decline in parental support that can occur with age, it has been consistently demonstrated as a significant source of support relevant to mental health throughout adolescence [20]. Moreover, parental support can compensate for low support from other sources [24]. Additionally, while certain parenting practices may have varying effects across cultures on adolescent mental health outcomes, the influence of parental support tends to be culturally universal [25].

Another important parenting practice associated with adolescent health is parental knowledge [26, 27]. Parental knowledge is commonly assessed by measuring parents’ awareness of their child’s whereabouts, activities and friends. Some prior research interpreted these findings as a positive effect of parental control and monitoring on youth outcomes, while others showed consistent evidence that parental knowledge is more likely to reflect voluntary youth disclosure [28, 29] and can, therefore, be indicative of the quality of the parent-adolescent relationship and better adolescent adjustment in general [27]. Parental knowledge generally tends to decrease as adolescents grow older [30], and while some studies reported consistent associations between parental knowledge and adolescent mental health problems across the high school years [26], other research demonstrated that parental knowledge was inversely related to health complaints for younger but not older adolescents [30]. Furthermore, the effect of different sources of parental knowledge can vary across cultures [31].

Parental rule-setting stands as another significant parenting practice promoting adolescent development [17]. While previous studies have provided considerable evidence for the protective effect of parental support, the impact of parental rule-setting on adolescent mental health remains somewhat uncertain and may differ based on cultural context [32]. Some findings suggested that although parental control enhances behavioural regulation in young people, it may restrict successful individuation, particularly during middle and late adolescence when adolescents seek greater independence and autonomy [33]. Accordingly, a high level of parental control has been found to be associated with poor adolescent mental health outcomes [14, 34], while the promotion of autonomy tends to enhance adolescent development [35], especially within a family context where adolescents feel close and attached to their parents [36]. Other studies have demonstrated that parental behavioural control may be beneficial for adolescents’ development, both in general [37] and when combined with parental knowledge [38]. Therefore, research on specific age and cultural groups is relevant for enhancing the understanding of the role of various parenting practices in different contexts.

### The Current Study

While many prior studies of parenting practices and adolescent psychosomatic complaints have relied on cross-sectional data [21, 22, 39], the importance of longitudinal studies focusing on parenting practices and youth problems has been emphasised [28]. Among the available longitudinal studies, most have investigated the association between independent variable(s) at baseline and dependent variable(s) at follow-up(s) whilst controlling for confounders [40, 41].

We extend previous research by using information on parenting practices and psychosomatic complaints from two time points, applying two complementary methods for analysing panel data. Using the first difference (FD) approach [42, 43], which represents the regression of the first difference (change over time) of one variable on the first difference (change over time) of another variable and offers insights about parallel temporal developments, we explore the associations between changes in parenting practices and changes in psychosomatic complaints. Additionally, we employ the lagged dependent variable (LDV) approach to examine the prospective associations, conducting regression analysis with controls for the initial value of the outcome variable. By using both approaches, we aim to achieve a more comprehensive understanding of the temporal aspects of the links between parenting practices and psychosomatic complaints.

In addition to age, previous studies have indicated that parenting practices vary by adolescents’ gender [44, 45], and considering gender is crucial for understanding the link between social relationships and health during adolescence [46]. Therefore, gender differences are examined throughout. Furthermore, prior inquiry has shown that both parenting practices and adolescent health complaints are associated with family structure [47], parental education [48] and parental country of birth [49], indicating that these characteristics should be adjusted for.

The aim of the present study was to investigate adolescents’ perceptions of parental support, knowledge, and rule-setting during middle and late adolescence, and to explore the associations between these parenting practices and adolescent psychosomatic complaints. The research questions were:

1) How do adolescents perceive their parents’ support, knowledge, and rule-setting across middle and late adolescence?
2) Are these parenting practices cross-sectionally associated with psychosomatic complaints?
3) Are changes in parenting practices associated with changes in psychosomatic complaints?
4) Are there prospective associations between parenting practices and psychosomatic complaints?
5) Are there gender differences?

## Methods

### Study Design and Material

**FIGURE 1:**
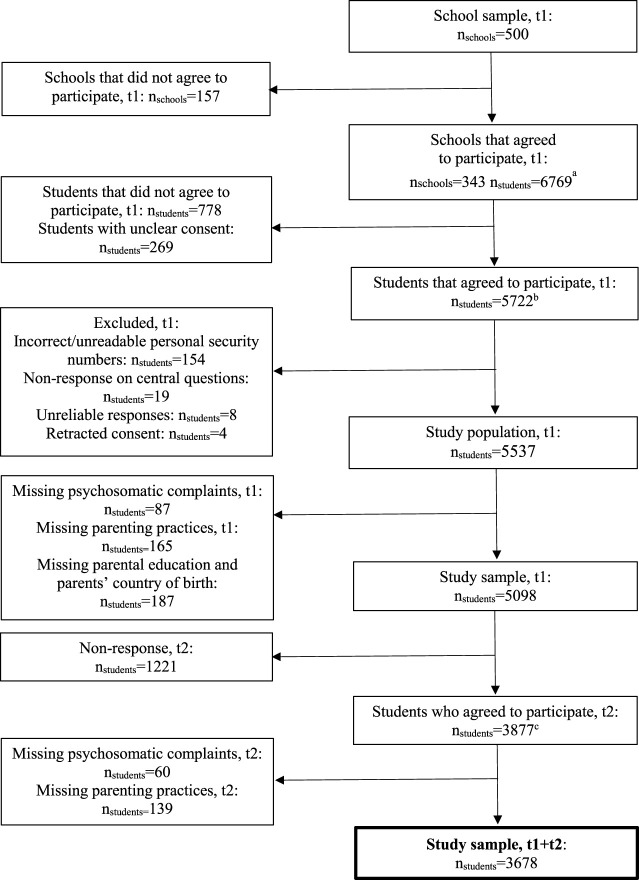
Flow chart of the study sample. Futura01, Sweden, 2017 and 2019. ^a^Present at school on the day of the classroom survey. ^b^Responded to the classroom survey. ^c^Responded to the web survey (83%) or the postal survey (17%).

The study used data from Futura01, a Swedish national cohort of individuals who attended grade 9 in 2017, most of whom born in 2001. Statistics Sweden randomly selected 500 schools and one class from each school. Participants completed self-report questionnaires at two time points: in 2017 (at age ∼15–16 years, T1) and in 2019 (∼17–18 years, T2). The first wave took place in schools and the students completed a paper-and-pencil questionnaire [50]. The second wave was conducted through a web and postal survey. Administrative register data, including parental education and country of birth, have been linked to the survey data. After excluding participants with missing information on any of the variables used in the study, the final sample amounted to 3,678 complete cases. Figure 1 provides more detailed information about the study sample.

Ethical approval has been provided by the Swedish Ethical Review Authority (ref. no. 2021-06504-01; 2022-02781-02; 2022-06502-02). Written informed consent was obtained from all study participants in the study.

### Measurements

Parenting practices were assessed using six statements, with two statements for each of the three scales: parental support, parental knowledge, and parental rule-setting. Parental support was measured with the statements: “I can easily get warmth and caring from my mother and/or father” and “I can easily get emotional support from my mother and/or father”. The statements for parental knowledge were: “My parent(s) know who I am with in the evenings” and “My parent(s) know where I am in the evenings.” Parental rule-setting was assessed with the statements “My parent(s) set definite rules about what I can do at home,” and “My parent(s) set definite rules about what I can do outside the home.” For each statement, there were five response alternatives: 5 = “Almost always,” 4 = “Often,” 3 = “Sometimes,” 2 = “Seldom,” and 1 = “Almost never.” Composite measures were created for the three scales by calculating mean scores based on answers to each pair of statements [51, 52]. Each scale consequently represented an overall score for mother and/or father parenting practices, which is a common approach in parenting research [53, 54]. Internal consistency was high (Cronbach’s alpha for parental rule-setting: T1 = 0.75, T2 = 0.78; parental knowledge: T1 = 0.76, T2 = 0.80; parental support: T1 = 0.89, T2 = 0.93). The pairwise correlations between the scales at each time point were weak to moderate (ranging from r = 0.03 to r = 0.40).

Psychosomatic complaints were measured via the question: “During the past 6 months, how often have you had…” and the complaints “stomach ache,” “difficulties falling asleep,” and “headache.” The response options were 5 = “Every day,” 4 = “A few times a week,” 3 = “Once a week,” 2 = “A few times a month” and 1 = “Less often or never.” The same items have previously been used to measure psychosomatic complaints [22]. A summative index combining the number and the frequency of complaints with a range of 3–15 was constructed, with higher values indicating more frequent and co-occurring complaints. The index showed acceptable internal consistency (Cronbach’s alpha: T1 = 0.65, T2 = 0.62).

Gender and birth year were based on information from the participants’ personal security numbers. Family structure was derived from survey information at T1 and T2, whereas parental education (highest level among parents) and parental country of birth were obtained from official registries.

### Analytical Strategy and Statistical Methods

Parenting practices and psychosomatic complaints were examined with descriptive statistics. Changes in parenting practices and in psychosomatic complaints were assessed with paired t-tests and with calculation of degree of change (∆ = T2-T1). Gender differences in mean values and in degree of change were examined with unpaired t-tests. Effect sizes were assessed with Cohen’s d. To evaluate the associations between parenting practices and psychosomatic complaints, linear regression analyses were performed. Cross-sectional associations were assessed for T1 and T2, respectively. To explore whether changes in parenting practices (T2-T1) were associated with changes (T2-T1) in adolescent psychosomatic complaints, we utilised the FD method for analysing panel data, regressing changes in the dependent variable (∆Y = T2-T1) on changes in the independent variables (∆X = T2-T1) (over 2 years) [43, 55]. An advantage of this method is that it reduces omitted variable bias by taking time-invariant confounding into account [42, 43, 56]. To examine the prospective associations, we applied the LDV method, i.e., regression analysis controlling for the initial value of the outcome variable. This method can be used as an additional analysis along with and, more specifically, following the FD method; accordingly, the associations that were found to be statistically significant in the FD analysis were tested with the LDV method [55]. It is worth highlighting that both these methods have pros (e.g., the FD method “provides a better safeguard against omitted variable bias” [43] (p. 938), while the LDV method allows testing temporal precedence) and cons (e.g., the FD approach bears a greater risk of type II error if the stability of change scores is too high, while the LDV approach bears a greater risk of type I error). They also provide information on different aspects of the associations, and by using both approaches we aim to explore the relationships between the variables of interest more thoroughly. To examine gender differences and to evaluate the combined effects of different parenting practices, interactions were tested. Robust standard errors were estimated in order to take the clustering of students in classes at T1 into account.

## Results

The study sample contained 3,678 adolescents, of whom 1,641 boys (44.6%) and 2037 girls (55.4%). Distributions of the covariates are presented in the Supplementary Table SA1.

Table 1 presents mean values of parenting practices and psychosomatic complaints, for all and by gender, and reports on differences by gender and across time. Distributions of the specific items are presented in the Supplementary Table SA2. The highest mean values were seen for parental support and parental knowledge, and the lowest mean value for parental rule-setting. There was no gender difference in perceived parental support, and parental support slightly decreased across time in boys and girls alike (∆ = -0.12 in boys, ∆ = −0.09 in girls, *p* = 0.283). Girls reported higher levels of parental knowledge than boys (at T1 and T2, *p* < 0.001). A minor decrease in parental knowledge across time was observed only in boys (*p* = 0.015), but there was no statistically significant gender difference in the degree of change (∆ = −0.06 in boys, ∆ = −0.02 in girls, *p* = 0.226). There was no gender difference in perceived parental rule-setting at T1; however, girls perceived slightly less rules from parents at T2 (*p* = 0.001). As seen in Supplementary Table SA2, there were gender differences in each of the two items of the parental rule-setting scale at T1 (parental rules at home and outside the home, respectively) and calculation of the mean scale’s score eliminated these differences. Mean values on the psychosomatic complaints’ summary index were higher in girls than in boys at both time points (*p* < 0.001) and increased equally for boys and girls from T1 to T2 (∆ = 0.21 in boys, ∆ = 0.17 in girls, *p* = 0.659).

**TABLE 1:** Mean values, standard deviations, and p-values from t-tests and Cohen’s d for gender and time differences in parenting practices and psychosomatic complaints. T1: 15–16 years, T2: 17–18 years. Futura01, Sweden, 2017 and 2019.

	All (*n* = 3678)				Boys (*n* = 1641)				Girls (*n* = 2037)								
	Mean (SD)	p time^a^	Effect size^b^	∆ (SD)^c^	Mean (SD)	p time^a^	Effect size^b^	∆ (SD)^c^	Mean (SD)	p time^a^	Effect size^b^	∆ (SD)^c^	p gender^d^	Effect size^e^	p ∆ gender^d^	Effect size^e^	Range^f^
Parental support																	1–5
T1	4.43 (0.90)	<0.001	**−0.11**	−0.10 (0.95)	4.44 (0.87)	<0.001	**−0.13**	−0.12 (0.97)	4.42 (0.91)	<0.001	**−0.09**	−0.09 (0.94)	0.536	0.02	0.283	−0.04	
T2	4.32 (0.97)				4.32 (0.97)				4.33 (0.98)				0.630	−0.02			
Parental knowledge																	1–5
T1	4.45 (0.79)	0.011	−0.05	−0.04 (0.94)	4.34 (0.84)	0.015	−0.07	−0.06 (1.01)	4.55 (0.74)	0.243	−0.03	−0.02 (0.88)	<0.001	**−0.27**	0.226	−0.04	
T2	4.41 (0.87)				4.28 (0.94)				4.53 (0.80)				<0.001	**−0.29**			
Parental rule-setting																	1–5
T1	3.07 (1.07)	<0.001	**−0.34**	−0.37 (1.15)	3.09 (1.07)	<0.001	**−0.29**	−0.32 (1.16)	3.06 (1.07)	<0.001	**−0.38**	−0.42 (1.14)	0.510	0.02	0.009	**0.09**	
T2	2.70 (1.12)				2.77 (1.14)				2.64 (1.11)				0.001	**0.11**			
Psychosomatic complaints																	
T1	7.06 (2.75)	<0.001	**0.07**	0.19 (2.46)	6.14 (2.43)	<0.001	**0.09**	0.21 (2.40)	7.80 (2.78)	0.002	**0.06**	0.17 (2.51)	<0.001	**−0.63**	0.659	0.01	3–15
T2	7.25 (2.72)				6.35 (2.43)				7.97 (2.74)				<0.001	**−0.62**			

^a^ Paired t-test for differences over time (between T1 and T2).

^b^ Cohen’s d effect size: T2 as reference group (positive effect sizes denote higher mean values at T2; negative effect sizes denote higher mean values at T1). Bold values denote statistical significance based on the 95% confidence intervals (CI).

^c^ Degree of change (∆= mean value at T2—mean value at T1).

^d^ Unpaired t-test for gender differences in mean values.

^e^ Cohen’s d effect size: boys as reference group (positive effect sizes denote higher mean values for boys; negative effect sizes denote higher mean values for girls). Bold values denote statistical significance based on the 95% confidence intervals (CI).

^f^ Range of scales (minimum—maximum).

Results from the cross-sectional regression analyses are displayed in Table 2. Higher levels of parental support were associated with fewer psychosomatic complaints in all models at both T1 and at T2. Parental knowledge was also significantly and inversely associated with psychosomatic complaints in all models except for Model 2 at T2 (but borderline significant, *p* = 0.057). Higher levels of parental rule-setting were associated with more psychosomatic complaints across all models (except for the crude model at T2); the associations were weaker than for the other parenting practices. Interactions between parenting practices and gender were tested (not presented in Table). One statistically significant interaction term was found, between gender and parental support at T1, showing that the inverse association between parental support and psychosomatic complaints was stronger for girls than for boys. Furthermore, a statistically significant interaction between parental knowledge and parental support was found at T1, signifying that the inverse association between parental knowledge and psychosomatic complaints was stronger for adolescents with higher values of parental support (not presented in Table).

**TABLE 2:** Results from cross-sectional analyses of psychosomatic complaints by parenting practices at T1 (15–16 years) and at T2 (17–18 years). Coefficients from linear regressions and 95% confidence intervals. *n* = 3,678. Futura01, Sweden, 2017 and 2019.

	Psychosomatic complaints (T1)					
	Crude^a^		Model 1^b^		Model 2^c^	
	b	95% CI	b	95% CI	b	95% CI
Parental support (T1)	−0.59	−0.69; −0.48	−0.52	−0.63; −0.41	−0.49	−0.60; −0.39
Parental knowledge (T1)	−0.45	−0.56; −0.34	−0.28	−0.39; −0.16	−0.24	−0.36; −0.13
Parental rule-setting (T1)	0.09	0.00; 0.17	0.12	0.04; 0.20	0.14	0.06; 0.22

	Psychosomatic complaints T2					
	Crude^a^		Model 1^b^		Model 2^c^	
	b	95% CI	b	95% CI	b	95% CI
Parental support (T2)	−0.51	−0.59; −0.42	−0.46	−0.55; −0.37	−0.44	−0.53; −0.34
Parental knowledge (T2)	−0.34	−0.43; −0.24	−0.14	−0.24; −0.04	−0.10	−0.21; 0.00
Parental rule-setting (T2)	0.07	−0.01; 0.14	0.09	0.01; 0.16	0.09	0.02; 0.16

^a^ Includes one independent variable at a time, adjusting for gender.

^b^ Mutually adjusts for all parenting practices and gender.

^c^ Mutually adjusts for all parenting practices, gender, family structure (T1/T2), parental education and parental country of birth.

The results of the FD analysis in Table 3 show that increases in parental support and in parental knowledge (but not in parental rule-setting) were associated with corresponding decreases in psychosomatic complaints. While the effect of change in parental support remained stable across models, the estimate for change in parental knowledge was attenuated when mutually adjusting for all parenting practices in Model 1. Adjusting additionally for family structure, parental education and parental country of birth in Model 2 did not affect the estimates.

**TABLE 3:** Results from analyses of change scores in psychosomatic complaints (T2-T1) by change in parenting practices (T2-T1) (first difference method). Coefficients from linear regressions and 95% confidence intervals. T1: 15–16 years, T2: 17–18 years. *n* = 3,678. Futura01, Sweden, 2017 and 2019.

	Change in psychosomatic complaints (T2-T1)					
	Crude^a^		Model 1^b^		Model 2^c^	
	b	95% CI	b	95% CI	b	95% CI
Change in parental support (T2-T1)	−0.33	−0.43; −0.23	−0.30	−0.40; −0.20	−0.30	−0.40; −0.20
Change in parental knowledge (T2-T1)	−0.19	−0.28; −0.09	−0.10	−0.19; −0.01	−0.10	−0.20; −0.01
Change in parental rule-setting (T2-T1)	−0.03	−0.10; 0.05	−0.02	−0.10; 0.05	−0.03	−0.10; 0.05

^a^ Includes one independent variable at a time, adjusting for gender.

^b^ Mutually adjusts for change in all parenting practices and gender.

^c^ Mutually adjusts for change in all parenting practices, gender, family structure (T1), parental education and parental country of birth.

Table 4 displays the results of the LDV analyses, with parenting practices at T1 predicting psychosomatic complaints at T2, adjusting for the baseline value of psychosomatic complaints. The results did not show any significant association between parenting practices at T1 and psychosomatic complaints at T2. Interaction terms by gender were tested, but none of these was statistically significant (not presented in Table).

**TABLE 4:** Results from analyses of psychosomatic complaints at T2 by parenting practices at T1, controlling for psychosomatic complaints at T1 (lagged dependent variable method). Coefficients from linear regressions and 95% confidence intervals. T1: 15–16 years, T2: 17–18 years. *n* = 3,678. Futura01, Sweden, 2017 and 2019.

	Psychosomatic complaints (T2)					
	Crude^a^		Model 1^b^		Model 2^c^	
	b	95% CI	b	95% CI	b	95% CI
Parental support (T1)	−0.06	−0.15; 0.02	−0.06	−0.14; 0.03	−0.04	−0.13; 0.05
Parental knowledge (T1)	−0.05	−0.15; 0.04	−0.04	−0.14; 0.05	−0.03	−0.13; 0.07
Psychosomatic complaints (T1)	0.55	0.52; 0.58	0.54	0.52; 0.57	0.54	0.51; 0.56

^a^ Includes one parenting practice, adjusting for psychosomatic complaints at T1 and gender.

^b^ Mutually adjusts for all parenting practices, psychosomatic complaints at T1 and gender.

^c^ Mutually adjusts for all parenting practices, psychosomatic complaints at T1 and gender, family structure (T1), parental education and parental country of birth.

Results from gender-stratified analyses of parenting practices and psychosomatic complaints are presented in the Supplementary Table SA3. Although there were some differences in the estimates and the levels of significance, the directions of almost all associations were similar for both genders.

## Discussion

The present study examined adolescent boys’ and girls’ perceptions of parental support, knowledge, and rule-setting across middle and late adolescence and the associations between these parenting practices and adolescent psychosomatic complaints, as well as potential gender differences.

The mean values indicated that a majority of adolescents were able to get support from their parents and that their parents were aware of who they spent their evenings with and their whereabouts. The most substantial change over age among the studied parenting practices was the decline in parental rule-setting, followed by a smaller decrease in parental support. These findings align with those from prior research reporting decreases in both parental rule-setting and support across middle and late adolescence [57].

The cross-sectional analyses of parenting practices and psychosomatic complaints showed that higher levels of parental support and knowledge were associated with lower levels of psychosomatic complaints, whereas higher levels of parental rule-setting were linked with more complaints. The analyses of changes across middle and late adolescence indicated that increases in parental support and parental knowledge were accompanied by decreases in psychosomatic complaints. However, no significant long-term effects of parental support or knowledge on psychosomatic complaints were found when assessing the predictive relationship between parenting practices at T1 and psychosomatic complaints at T2 while controlling for psychosomatic complaints at T1. Taken together, the results indicate that parenting practices are linked with concurrent psychosomatic complaints, but do not exhibit any long-term effects on this specific outcome. Our findings are in line with those of an Australian study [58] which showed that parenting was associated with subjective wellbeing in real time but not in future time points. This suggests that positive parenting practices cannot be assumed to be ‘banked’ by adolescents, but that parental involvement has to be frequent and ongoing [58]. It is, however, important to acknowledge that the lack of clear associations in the prospective analyses could partly be attributed to the relatively long interval between the two time points, and that more frequent repeated measurements could have yielded different results [59].

Parental support emerged as the most important of all studied parenting practices in relation to adolescents’ psychosomatic complaints. This finding aligns with previous research indicating the protective role of parental support in relation to the mental and psychosomatic health of adolescents [20, 21, 23]. The inverse association between parental support and psychosomatic complaints in the cross-sectional analyses and the analyses of change may be understood as parental support providing psychological resources that enhance an individual’s ability to cope with stress [60]. Furthermore, parental support fosters a sense of belonging through acceptance and inclusion, thereby promoting self-esteem and self-regulation in young people [61, 62].

Parental knowledge was also connected with psychosomatic complaints in the cross-sectional analyses and the analyses of change, although the associations were attenuated when parental support was included in the models. While parental support and knowledge were cross-sectionally correlated, additional analyses (not presented) also indicated a prospective association between parental support at T1 and knowledge at T2, but not the other way around. This finding aligns with previous research suggesting that parental warmth and support act as prerequisites for young people’s disclosure, leading to higher parental knowledge [63, 64], emphasising the role of parental support.

The items used to measure parental knowledge were based on adolescents’ reports of their parents’ awareness of their daily activities, which is the most common operationalisation of parental monitoring [28]. This operationalisation has however been criticised, and it has been suggested that parents’ knowledge of their children’s activities may not necessarily reflect active monitoring efforts but rather the willingness of adolescents to disclose information [28]. Furthermore, a longitudinal study by Stattin & Kerr [29] conducted in a Swedish context supported this interpretation. Given these considerations, it is assumed in this study that the measure of parental knowledge primarily reflects youth disclosure of information rather than parental monitoring efforts. However, since the measure does not distinguish between different sources of parental knowledge, the specific phenomenon captured and the underlying mechanisms can only be speculated upon.

The positive association between parental rule-setting and psychosomatic complaints in the cross-sectional analyses was small but consistent in the adjusted models. One interpretation is that as adolescents go through processes of separation and individuation and gain more autonomy, having excessive rules and restrictions may make them feel incompetent and less confident in themselves. For example, a study by Gittins & Hunt [65] found that parental rules can increase self-criticism and undermine self-beliefs in 12–14-year-olds, and this effect may be even stronger in older ages when the need for independence becomes more pronounced. Another interpretation is linked to the evidence indicating that adolescents may perceive parental control in varying ways based on the social domain of the topic parents attempt to regulate (thereby, depending on the particular behaviours being controlled) [66]. As this study assessed a narrow range of behaviours, the generalisation of the findings to other contexts of parental rule-setting should be made with caution. Furthermore, the small coefficients of parental rule-setting in the cross-sectional analyses and the lack of associations in the analyses of changes indicate that the other studied parenting practices play a more significant role for adolescent psychosomatic complaints.

While there were some gender differences in perceived parenting practices across middle and late adolescence, the links between parenting practices and psychosomatic complaints were generally similar among boys and girls. One exception was the stronger association between parental support and psychosomatic complaints at T1 among girls, reflecting findings from previous studies [21]. As discussed above, self-esteem is one mechanism linking social support [67], and specifically, parental support [62], to health. A large body of research has consistently reported lower self-esteem in girls compared to boys [68, 69]. Moreover, self-esteem is inversely associated with mental health problems [69] and contributes to explaining the higher prevalence of psychosomatic complaints in girls [70]. Therefore, girls may particularly benefit from parental support through sustaining their identity and self-esteem.

A key strength of the current study is the use of data from a large national cohort, with identical measures on parenting practices and psychosomatic complaints at two time points. Notwithstanding, there are also limitations. Firstly, even though the response rate was relatively high, attrition at the school level at T1 and at the individual level at both T1 and T2 may have restricted the generalisability of the findings. Another limitation relates to the measurements of parenting practices. The measurement of each parenting practice was based on only two items. Since they were not assessed separately for mothers and for fathers, we were also unable to scrutinise potential differences between mothers’ and fathers’ parenting practices in relation to adolescents’ psychosomatic complaints. Furthermore, due to the nature of the parental knowledge measurement, it was not possible to determine which construct was captured (youth’s disclosure or parental monitoring), and therefore the results regarding parental knowledge should be interpreted with caution. Additionally, the measure of psychosomatic complaints consisted of only three items with limited internal consistency. Future studies into the links between parenting practices and psychosomatic complaints using more comprehensive measures, e.g., including subscales of different types of complaints, are recommended.

### Conclusion

The findings of the present study emphasise the importance of ongoing parenting practices during middle and late adolescence. While increases in parental support and parental knowledge from middle to late adolescence were linked to reductions in psychosomatic complaints, these practices did not show prospective associations with adolescent psychosomatic complaints. This underscores the importance of consistent and ongoing parental engagement, particularly in terms of providing support to adolescents. The results suggest that interventions aimed at enhancing parental support during this age period are likely to have an impact on adolescent mental health.
