# Supplementary material for: Parenting Practices and Psychosomatic Complaints Among Swedish Adolescents
Source: Int J Public Health. 2023 Dec 21;68:1606580. doi: 10.3389/ijph.2023.1606580 (PMC10766014; doi:10.3389/ijph.2023.1606580)
Supplement: Supplementary file 1 [file DataSheet1.docx]

**Supplementary material**

**Appendix Table SA1.** Distribution of sociodemographic characteristics, for all and by gender. T1: 15-16 years,
T2: 17-18 years. Futura01, Sweden, 2017 and 2019.

|  | All  (n=3678) | Boys (n=1641) | Girls (n=2037) |
| --- | --- | --- | --- |
|  | % | % | % |
| Family structure |  |  |  |
| Lives with both parents |  |  |  |
| T1 | 70.5 | 71.7 | 69.5 |
| T2 | 63.3 | 65.2 | 61.7 |
| Shared residence |  |  |  |
| T1 | 13.5 | 14.2 | 13.0 |
| T2 | 11.2 | 11.1 | 11.2 |
| Lives with single parent |  |  |  |
| T1 | 13.8 | 12.0 | 15.3 |
| T2 | 15.7 | 14.2 | 16.8 |
| Lives in own accommodation |  |  |  |
| T1 | - | - | - |
| T2 | 2.8 | 2.7 | 2.9 |
| Other |  |  |  |
| T1 | 2.2 | 2.1 | 2.3 |
| T2 | 7.2 | 6.8 | 7.4 |
|  |  |  |  |
| Parental education |  |  |  |
| Upper secondary school ≤2 years | 15.3 | 14.0 | 16.3 |
| Upper secondary school ≥3 years | 20.6 | 20.5 | 20.6 |
| Tertiary education ≤2 years | 17.9 | 17.7 | 18.2 |
| Tertiary education ≥3 years | 46.2 | 47.8 | 44.9 |
|  |  |  |  |
| Parental country of birth |  |  |  |
| At least one parent born in Sweden | 84.8 | 86.0 | 83.9 |
| At least one parent born in Europe | 4.9 | 4.3 | 5.4 |
| Two parents born outside Europe | 10.3 | 9.7 | 10.7 |

**Appendix Table SA2.** Distribution of parenting practices and psychosomatic complaints and differences by gender assessed with χ^2^ tests. T1: 15-16 years, T2: 17-18 years. Futura01, Sweden, 2017 and 2019.

|  | All (n=3678) | | | | | Boys (n=1641) | | | | | Girls (n=2037) | | | | | p gender |
| --- | --- | --- | --- | --- | --- | --- | --- | --- | --- | --- | --- | --- | --- | --- | --- | --- |
| Parenting practices | Almost never | Seldom | Some-times | Often | Almost always | Almost never | Seldom | Some-times | Often | Almost always | Almost never | Seldom | Some-times | Often | Almost always |  |
|  | % | % | % | % | % | % | % | % | % | % | % | % | % | % | % |  |
| Parental support |  |  |  |  |  |  |  |  |  |  |  |  |  |  |  |  |
| I can easily get warmth and caring from my mother and/or father |  |  |  |  |  |  |  |  |  |  |  |  |  |  |  |  |
| T1 | 1.3 | 2.5 | 8.9 | 18.7 | 68.6 | 1.5 | 2.0 | 8.5 | 20.4 | 67.6 | 1.1 | 2.9 | 9.2 | 17.3 | 69.5 | 0.045 |
| T2 | 2.2 | 4.0 | 9.8 | 21.1 | 62.9 | 2.1 | 4.2 | 10.2 | 21.5 | 62.0 | 2.2 | 3.8 | 9.6 | 20.8 | 63.6 | 0.843 |
| I can easily get emotional support from my mother and/or father |  |  |  |  |  |  |  |  |  |  |  |  |  |  |  |  |
| T1 | 2.9 | 4.5 | 9.8 | 21.0 | 61.8 | 2.8 | 3.5 | 10.3 | 21.4 | 62.0 | 3.0 | 5.4 | 9.4 | 20.7 | 61.5 | 0.083 |
| T2 | 3.1 | 5.2 | 11.8 | 22.3 | 57.6 | 2.7 | 5.6 | 12.2 | 22.0 | 57.5 | 3.3 | 4.9 | 11.6 | 22.5 | 57.7 | 0.658 |
| Parental knowledge |  |  |  |  |  |  |  |  |  |  |  |  |  |  |  |  |
| My parent(s) know who I am with in the evenings |  |  |  |  |  |  |  |  |  |  |  |  |  |  |  |  |
| T1 | 2.1 | 3.2 | 8.4 | 23.5 | 62.9 | 3.0 | 4.5 | 9.7 | 27.2 | 55.6 | 1.4 | 2.1 | 7.2 | 20.5 | 68.8 | <0.001 |
| T2 | 3.1 | 4.0 | 8.5 | 21.9 | 62.5 | 4.1 | 5.0 | 10.9 | 24.9 | 55.1 | 2.3 | 3.1 | 6.6 | 19.5 | 68.5 | <0.001 |
| My parent(s) know where I am in the evenings |  |  |  |  |  |  |  |  |  |  |  |  |  |  |  |  |
| T1 | 1.2 | 2.6 | 8.1 | 22.2 | 65.9 | 1.4 | 3.5 | 9.1 | 26.3 | 59.7 | 1.0 | 1.9 | 7.2 | 19.0 | 70.9 | <0.001 |
| T2 | 1.8 | 3.6 | 7.5 | 20.9 | 66.2 | 2.5 | 4.8 | 9.2 | 24.3 | 59.2 | 1.2 | 2.7 | 6.2 | 18.1 | 71.8 | <0.001 |
| Parental rule-setting |  |  |  |  |  |  |  |  |  |  |  |  |  |  |  |  |
| My parent (s) have definite rules for what I can do at home |  |  |  |  |  |  |  |  |  |  |  |  |  |  |  |  |
| T1 | 11.5 | 20.9 | 29.7 | 24.7 | 13.2 | 9.8 | 19.1 | 28.2 | 27.8 | 15.1 | 12.9 | 22.4 | 30.9 | 22.2 | 11.6 | <0.001 |
| T2 | 20.5 | 25.2 | 26.0 | 18.5 | 9.8 | 17.5 | 22.9 | 26.1 | 21.6 | 11.9 | 22.8 | 27.1 | 25.9 | 16.1 | 8.1 | <0.001 |
| My parent (s) have definite rules for what I can do outside the home |  |  |  |  |  |  |  |  |  |  |  |  |  |  |  |  |
| T1 | 10.7 | 22.6 | 28.7 | 24.5 | 13.5 | 12.9 | 24.6 | 26.2 | 24.5 | 11.8 | 9.0 | 21.1 | 30.7 | 24.4 | 14.8 | <0.001 |
| T2 | 20.1 | 27.3 | 26.2 | 17.4 | 9.0 | 20.7 | 27.9 | 25.1 | 17.5 | 8.8 | 19.7 | 26.8 | 27.1 | 17.2 | 9.2 | 0.661 |
|  |  |  |  |  |  |  |  |  |  |  |  |  |  |  |  |  |
| Psychosomatic complaints | Less often or never | Some time a month | Once a week | A few times a week | Every day | Less often or never | Some time a month | Once a week | A few times a week | Every day | Less often or never | Some time a month | Once a week | A few times a week | Every day |  |
|  | % | % | % | % | % | % | % | % | % | % | % | % | % | % | % |  |
| Stomach ache |  |  |  |  |  |  |  |  |  |  |  |  |  |  |  |  |
| T1 | 32.6 | 40.4 | 13.2 | 11.7 | 2.1 | 50.4 | 33.9 | 8.8 | 6.0 | 0.9 | 18.2 | 45.6 | 16.8 | 16.3 | 3.1 | <0.001 |
| T2 | 32.1 | 37.9 | 13.8 | 13.6 | 2.6 | 50.5 | 31.9 | 9.5 | 6.7 | 1.4 | 17.2 | 42.7 | 17.3 | 19.2 | 3.6 | <0.001 |
| Headache |  |  |  |  |  |  |  |  |  |  |  |  |  |  |  |  |
| T1 | 27.9 | 33.2 | 16.6 | 17.3 | 5.0 | 38.8 | 35.5 | 14.1 | 9.8 | 1.8 | 19.1 | 31.4 | 18.6 | 23.4 | 7.5 | <0.001 |
| T2 | 26.0 | 34.1 | 17.2 | 18.1 | 4.6 | 37.0 | 38.4 | 14.1 | 9.0 | 1.5 | 17.1 | 30.7 | 19.6 | 25.5 | 7.1 | <0.001 |
| Difficulties falling asleep |  |  |  |  |  |  |  |  |  |  |  |  |  |  |  |  |
| T1 | 27.5 | 26.1 | 15.9 | 20.8 | 9.3 | 32.4 | 27.4 | 15.2 | 17.3 | 7.7 | 24.4 | 25.0 | 16.4 | 23.7 | 10.5 | <0.001 |
| T2 | 24.2 | 25.9 | 18.3 | 22.0 | 9.6 | 25.6 | 27.5 | 17.6 | 21.3 | 8.0 | 23.1 | 24.7 | 18.8 | 22.6 | 10.8 | 0.006 |

**Appendix Table SA3** Results from cross-sectional, analyses of change scores (FD method) and prospective analyses (LDV method) of parenting practices and psychosomatic complaints for boys (n=1641) and girls (n=2073) separately. T1: 15-16 years, T2: 17-18 years. Futura01, Sweden, 2017 and 2019.

| Cross-sectional analyses (T1)^a^ | Psychosomatic complaints (T1) | | | |
| --- | --- | --- | --- | --- |
|  | Boys | | Girls | |
|  | b | 95% CI | b | 95% CI |
| Parental support (T1) | -0.35 | -0.51; -0.19 | -0.59 | -0.74; -0.45 |
| Parental knowledge (T1) | -0.22 | -0.37; -0.06 | -0.27 | -0.42; -0.11 |
| Parental rule-setting (T1) | 0.09 | -0.03; 0.20 | 0.18 | 0.07; 0.29 |
|  |  |  |  |  |
|  |  |  |  |  |
| Cross-sectional analyses (T2)^b^ | Psychosomatic complaints (T2) | | | |
|  | Boys | | Girls | |
|  | b | 95% CI | b | 95% CI |
| Parental support (T2) | -0.50 | -0.64; -0.36 | -0.39 | -0.51; -0.26 |
| Parental knowledge (T2) | -0.14 | -0.29; 0.00 | -0.07 | -0.21; 0.08 |
| Parental rule-setting (T2) | 0.09 | -0.00; 0.19 | 0.10 | -0.02; 0.21 |
|  |  | |  | |
|  |  | |  | |
| FD method ^c^ | Change in psychosomatic complaints (T2-T1) | | | |
|  | Boys | | Girls | |
|  | b | 95% CI | b | 95% CI |
| Change in parental support (T2-T1) | -0.32 | -0.45; -0.18 | -0.29 | -0.42; -0.17 |
| Change in parental knowledge (T2-T1) | -0.03 | -0.16; 0.09 | -0.16 | -0.30; -0.03 |
| Change in parental rule-setting (T2-T1) | -0.01 | -0.12; 0.09 | -0.05 | -0.16; 0.06 |
|  |  |  |  |  |
|  |  |  |  |  |
| LDV method ^d^ | Psychosomatic complaints (T2) | | | |
|  | Boys | | Girls | |
|  | b | 95% CI | b | 95% CI |
| Parental support (T1) | -0.08 | -0.21; 0.04 | -0.00 | -0.13; 0.12 |
| Parental knowledge (T1) | -0.08 | -0.21; 0.05 | 0.02 | -0.14; 0.17 |
| Parental rule-setting (T1) | 0.07 | -0.03; 0.17 | 0.09 | -0.01; 0.18 |

^a^ Cross-sectional analyses of psychosomatic complaints at T1 by parenting practices at T1, mutually adjusting for all parenting practices, family structure (T1), parental education and parental country of birth.

^b^ Cross-sectional analyses of psychosomatic complaints at T2 by parenting practices at T2, mutually adjusting for all parenting practices, family structure (T2), parental education and parental country of birth.

^c^ FD method: Change in psychosomatic complaints (T2-T1) by change in parenting practices (T2-T1), mutually adjusting for change in all parenting practices (T2-T1), family structure (T1), parental education and parental country of birth.

^d^ LDV method: Psychosomatic complaints at T2 by parenting practices at T1, controlling for psychosomatic complaints at T1, family structure (T1), parental education and parental country of birth.
